# Evaluation of delirium frequency and factors affecting it in postanesthesia care unit: A prospective observational study

**DOI:** 10.1097/MD.0000000000050668

**Published:** 2026-09-11

**Authors:** Muhammed Emin Yildirim, Volkan Hanci, Duriye Gul Inal, Hale Aksu, Sule Ozbilgin, Semih Kucukguclu

**Affiliations:** a Department of Anesthesiology and Reanimation, Sancaktepe Sehit Prof. Dr. Ilhan Varank Training and Research Hospital, University of Health Sciences, Istanbul, Turkey; b Department of Anesthesiology and Reanimation, Faculty of Medicine, Dokuz Eylul University, Izmir, Turkey.

**Keywords:** delirium, pain, postanesthesia care unit, postoperative, risk factors

## Abstract

Postoperative delirium (POD) is a common but often underrecognized complication after surgery and is associated with increased morbidity and prolonged hospitalization. This study aimed to determine the incidence of POD in the postanesthesia care unit (PACU) and to identify perioperative factors independently associated with its development. This prospective observational study included adult surgical patients admitted to the PACU between August 2021 and January 2023. Delirium assessments were performed daily using the confusion assessment method for the intensive care unit, nursing delirium screening scale, and intensive care delirium screening checklist after extubation and discontinuation of continuous sedative infusions. Demographic characteristics, comorbidities, intraoperative variables, and postoperative biochemical parameters were recorded. Independent predictors of POD were determined using multivariable logistic regression. A total of 314 patients were analyzed, and POD occurred in 22.6% of cases, predominantly hypoactive in subtype (76%). Independent predictors included age ≥65 years (odds ratio [OR]: 3.08; 95% confidence interval [CI]: 1.10 to 8.63; *P* = .032), higher PACU pain scores (OR: 1.28; 95% CI: 1.03 to 1.59; *P* = .025), lower hematocrit (OR: 0.89; 95% CI: 0.82 to 0.98; *P* = .014), reduced glomerular filtration rate (OR: 0.98; 95% CI: 0.97 to 1.00; *P* = .014), and longer fentanyl infusion duration (OR: 1.09; 95% CI: 1.01 to 1.17; *P* = .025). Patients with delirium had significantly longer PACU stays, higher intensive care unit admission rates, prolonged hospitalizations, and increased postoperative complications and mortality. POD is frequent among PACU patients and is strongly associated with advanced age, impaired renal function, anemia, severe postoperative pain, and prolonged opioid exposure. Implementation of early structured screening and optimization of modifiable perioperative risk factors may help reduce delirium incidence and improve postoperative outcomes.

## 1. Introduction

Delirium is an acute organic brain dysfunction characterized by disturbances in consciousness, disorganized thinking, and impaired attention.^[1]^ It affects 10% to 24% of adult inpatients, 37% to 46% of surgical patients,^[2]^ and up to 60% to 80% of mechanically ventilated patients.^[1]^ Postoperative incidence varies substantially (9%–87%), influenced by age, comorbidities, assessment tools, and surgical type.^[1–3]^ While nondelirious patients generally return to baseline cognition within 1 month, recovery in those with delirium may take 6 to 12 months and is associated with increased dementia risk, more than a 2-fold prolongation of hospital stay, persistent functional dependence at 6 months, and an estimated 10% rise in mortality for each additional day of delirium.^[1,4]^

Delirium rates may reach 15% in postanesthesia care units (PACU) and 70% in intensive care units (ICU); however, 64% to 84% of cases remain unrecognized and 33% to 66% are entirely undiagnosed.^[3,5]^ The hypoactive subtype, observed in 20% to 50% of cases – particularly among older adults – is characterized by psychomotor slowing, somnolence, and confusion. Despite its association with increased mortality, it frequently goes unnoticed without systematic assessment.^[3,6]^ Because anesthesia and surgery impose substantial physiological stress on the brain, delirium observed in the PACU may represent an early manifestation of postoperative delirium and predict subsequent adverse outcomes, highlighting the need for early detection.^[1,4]^ Early postoperative delirium in the PACU may therefore signal emerging postoperative neurocognitive dysfunction and serve as a warning indicator for later complications, prolonged recovery, or increased mortality.

The primary objective of this study was to determine the incidence of postoperative delirium (POD) among patients admitted to the PACU and to identify perioperative risk factors associated with its development. Secondary objectives included evaluating the relationship between POD and postoperative complications, PACU and ICU length of stay, and mortality. We hypothesized that advanced age, higher American Society of Anesthesiologists (ASA) physical status, frailty, greater transfusion requirements, prolonged PACU stay, and abnormal laboratory parameters would independently predict POD.

## 2. Material and methods

### 2.1. Study design and ethical approval

This prospective observational study was conducted in the PACU of Dokuz Eylül University Faculty of Medicine between August 1, 2021, and January 31, 2023. The PACU functions as an intermediate intensive care setting that admits both intubated and extubated postoperative patients. Extubated patients are monitored for several hours until discharge criteria are met, whereas intubated or hemodynamically unstable patients may remain under prolonged observation. The length of PACU stay typically does not exceed 5 days, and no minimum duration was required for inclusion.

Ethical approval was obtained from the Dokuz Eylül University Faculty of Medicine Non-Interventional Research Ethics Committee (date: July 28, 2021; decision no: 2021/22-04; Chairperson: Prof Dr Mehmet Birhan Yilmaz). Written informed consent was obtained from all participants or their legal representatives.

### 2.2. Inclusion and exclusion criteria

All adult surgical patients (≥18 years) admitted to the PACU after elective or emergency procedures during the study period were screened. Inclusion required the ability to obtain informed consent. Patients were excluded if they had preexisting neurocognitive disorders (e.g., Alzheimer disease and dementia), which were identified through comprehensive preoperative medical history, electronic health record reviews, and interviews with patients or their primary caregivers; routine formal objective cognitive screening (such as mini-mental state examination or Montreal cognitive assessment) was not routinely performed prior to surgery. Additional exclusion criteria included a history of intracranial surgery, active COVID-19 infection at the time of surgery, incomplete baseline data, refusal or withdrawal of consent, or death prior to extubation or PACU discharge.

### 2.3. Delirium assessment

Delirium was assessed by a trained team consisting of the attending anesthesiologist, resident physician, and senior PACU nurse. Evaluations were performed daily, but only after extubation and discontinuation of sedative infusions. Patients who remained intubated or under continuous sedation were considered not assessable and were reevaluated following sedation weaning and extubation.

Because delirium symptoms may fluctuate over the course of the day, bedside nurses performed systematic screening using the nursing delirium screening scale (NuDESC) every 2 hours during routine patient care and repositioning. When a NuDESC score suggested possible delirium (NuDESC ≥ 1), the responsible anesthesiologist was immediately notified to perform formal confirmatory assessments using the confusion assessment method for the intensive care unit (CAM-ICU-7) and the intensive care delirium screening checklist (ICDSC). In cases where screening or diagnostic instrument results were discordant (e.g., positive NuDESC but negative CAM-ICU-7), an expert clinical evaluation conducted by the attending anesthesiologist based on diagnostic and statistical manual of mental disorders, 5th edition diagnostic criteria served as the ultimate diagnostic benchmark. Delirium was recorded as present if confirmed by CAM-ICU-7, ICDSC (score ≥ 4), or the expert diagnostic and statistical manual of mental disorders, 5th edition clinical assessment at any point during the PACU stay.

The Turkish version of the CAM-ICU demonstrated 65% to 69% sensitivity and 97% specificity (κ = 0.96) in the validation study by Akinci et al.^[7]^ The Turkish validation of ICDSC by Köse et al^[8]^ reported 73% to 87.7% specificity and 50% to 85.7% sensitivity for primary nurses, and 71.1% to 87.8% specificity and 75% to 100% sensitivity for ICU nurses. The NuDESC validation conducted by Çinar et al^[9]^ showed 92.27% sensitivity and 92.72% specificity. Scores from each tool were recorded separately, and the ultimate presence of delirium was determined based on this structured decision algorithm.

### 2.4. Data collection

Demographic variables (age and sex), ASA physical status, comorbidities, Charlson comorbidity index, metabolic equivalent of task (MET), fatigue, resistance, ambulation, illnesses, and loss of weight (FRAIL) index, and history of alcohol, drug, or tobacco use were recorded.

Perioperative data included anesthesia duration, anesthesia technique, induction agents, intraoperative hemodynamic events, inotrope use, perioperative medications (benzodiazepines, opioids, and ketamine), and blood product transfusions. Processed electroencephalographic depth of anesthesia monitoring (e.g., bispectral index) was not routinely applied across all surgical cases and was guided by standard clinical judgment, end-tidal gas monitoring, and hemodynamic stability. In the PACU, data collection included sedative and opioid administration, vital signs (temperature, oxygen saturation, and mean arterial pressure), laboratory values (blood gas, biochemistry, and hemogram), and analgesic requirements. All laboratory values used in the analyses corresponded to routine morning samples obtained on the day delirium was 1st assessed.

Baseline (preoperative) laboratory parameters were screened prior to surgery to confirm baseline organ function. Postoperative laboratory values obtained in the PACU were specifically analyzed in the multivariable model to capture acute perioperative physiological derangements (e.g., acute postoperative drop in hematocrit, glomerular filtration rate (GFR) impairment, and systemic inflammatory response indicated by C-reactive protein [CRP]) directly concurrent with the delirium assessment.

All intraoperative data – including anesthesia duration, administered fluids, blood products, medications, and hemodynamic events – were obtained from standardized anesthesia records. Perioperative information was collected prospectively from these records and contemporaneous PACU documentation. Postoperative findings, including laboratory results, consultation notes, and ward progress entries, were obtained from the hospital’s electronic medical record system to track complications and verify outcomes. These data were accessed in real time as part of routine clinical documentation rather than retrospective chart review. All variables were prospectively entered into a standardized study database.

Because the study was exploratory, all clinically relevant preoperative, intraoperative, and postoperative variables with potential relevance to postoperative delirium were collected. No single variable was defined as a primary predictor; instead, multivariable analysis was used to determine independent associations.

### 2.5. Postoperative complications

Postoperative complications were extracted using predefined objective criteria to reduce observer bias. Complications included wound infection (purulent discharge or positive swab culture), pneumonia (radiologic infiltrate with purulent sputum and positive culture), other pulmonary events, sepsis (systemic inflammatory response syndrome criteria plus positive culture), arrhythmias, atrial fibrillation, renal impairment (<0.5 mL/kg/h urine output), and other clinically significant events requiring intervention.

The duration of PACU, ICU, and hospital stay, as well as discharge status (alive or deceased), were recorded.^[10]^

### 2.6. Statistical analysis

Data were analyzed using SPSS version 24.0 (IBM Corp.). An a priori sample size estimation indicated that a minimum cohort of 280 patients was required to detect independent predictors of postoperative delirium, assuming an estimated PACU incidence of 20% (α = 0.05, power = 80%). The normality of continuous variables was assessed using Kolmogorov–Smirnov and Shapiro–Wilk tests. Continuous parameters are reported as median (interquartile range) and compared via the Mann–Whitney *U* test. Categorical variables are presented as counts (percentages) and evaluated using Pearson chi-square or Fisher exact test, as appropriate.

To identify independent risk factors for postoperative delirium while safeguarding against model overfitting and multicollinearity, a structured candidate selection strategy was applied for multivariable binary logistic regression. Candidate variables were selected based on clinical relevance, biological plausibility, and preliminary univariable associations (*P* < .10). Intraoperative candidate variables, including surgical and anesthesia duration, were evaluated during univariable screening. Variables demonstrating no statistically significant association with delirium (such as anesthesia duration, *P* > .05) were not retained in the final multivariable model. Multicollinearity was systematically evaluated using correlation matrices and variance inflation factors < 3. To prevent redundancy among collinear variables representing identical biological domains (e.g., hematocrit vs hemoglobin; creatinine vs GFR; and CRP vs CRP/albumin ratio), only the predictor demonstrating the most robust univariable fit within each domain was retained for the final multivariable model.

The multivariable model was constructed using backward stepwise elimination. Given the 71 observed delirium events, restricting the final model to 5 independent predictors yielded an events per variable ratio of 14.2, satisfying the recommended methodological standard (events per variable >10). Clinically plausible interaction terms (e.g., age × pain and anemia × transfusion) were tested but showed no model improvement. Model discrimination was evaluated using the area under the receiver operating characteristic curve (AUC), and calibration was assessed via the Hosmer–Lemeshow goodness-of-fit test. Adjusted odds ratios (ORs) with 95% confidence intervals (CIs) are reported. Two-sided *P* values <.05 were considered statistically significant.

The primary outcome was postoperative delirium occurring during the PACU stay. Secondary outcomes included postoperative complications (as a composite and individually), ICU and hospital length of stay, and 30-day all-cause mortality.

## 3. Results

**Figure 1.**
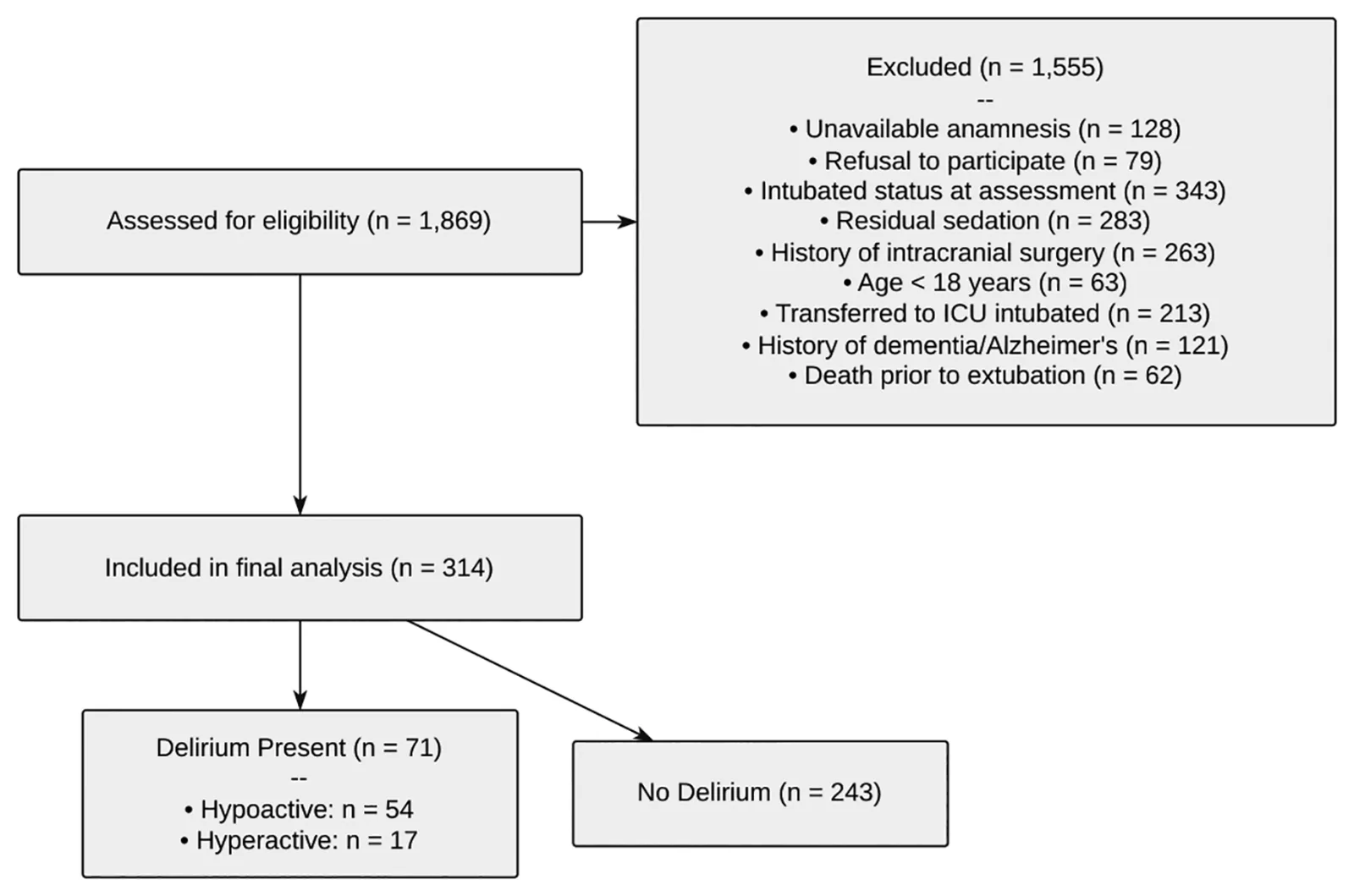
Flow diagram of patient recruitment, exclusion criteria, and postanesthesia care unit delirium assessment. ICU = intensive care unit.

A total of 1869 patients treated in the PACU of Dokuz Eylul University Faculty of Medicine between August 1, 2021 and January 31, 2023 were screened, and 314 met the eligibility criteria for inclusion in the final analysis. Exclusions were due to unavailable anamnesis (n = 128), refusal to participate (n = 79), intubated status at the time of assessment (n = 343), residual sedation (n = 283), history of intracranial surgery (n = 263), age under 18 years (n = 63), transfer to the ICU while intubated (n = 213), diagnosis of Alzheimer disease or dementia (n = 121), and death prior to extubation (n = 62) (Fig. 1). Demographic characteristics and their association with delirium are presented in Table 1, and comorbidities and surgical variables associated with delirium are shown in Table 2.

**Table 1:** The relationship between demographic characteristics of the patients and development of delirium.

	No delirium (n = 243)	Delirium present (n = 71)	*P*
Male	129 (53.1%)	31 (43.7%)	.162*
Female	114 (46.9%)	40 (56.3%)	
Under 65 yr old	101 (41.6%)	9 (12.7%)	**<.001** *
Over 65 yr old	142 (58.4%)	62 (87.3%)	
ASA 1–2	119 (49%)	25 (35.2%)	**.041** *
ASA 3–4	124 (51%)	46 (64.8%)	
MET <4	87 (35.8%)	38 (53.5%)	**.007** *
MET >4	156 (64.2%)	33 (46.5%)	
Non-frail	122 (50.2%)	22 (31%)	**.004** *
Frail	121 (49.8%)	49 (69%)	
Malnutrition is present	74 (30.5%)	28 (39.4%)	.155*
No malnutrition	169 (69.5%)	43 (60.6%)	
CCI	5 (3–7)	5 (4–7)	.141**
10 yr of survival	21% (0–77)	21% (0–53)	.09**

Significant results (*P* < .05) is shown in bold.

ASA = American Society of Anesthesiologists, CCI = Charlson comorbidity index, FRAIL = fatigue, resistance, ambulation, illnesses, and loss of weight index, MET = metabolic equivalent of task.

* Pearson chi-square.

** Mann–Whitney *U* test.

**Table 2:** Association of comorbidities and type of surgery with delirium development.

Comorbidity	Delirium in patients with n (%)	Delirium in patients without n (%)	*P*
Hypertension	54/187 (28.9%)	17/127 (13.4%)	**.001** *
Malignancy	23/128 (18.0%)	48/186 (25.8%)	.103*
Diabetes mellitus	23/102 (22.5%)	48/212 (22.6%)	.985*
Coronary artery disease	20/83 (24.1%)	51/231 (22.1%)	.706*
Heart failure	8/39 (20.5%)	63/275 (22.9%)	.738*
Atrial fibrillation	13/41 (31.7%)	58/273 (21.2%)	.135*
Lung disease	17/79 (21.5%)	54/235 (23.0%)	.788*
COPD	9/41 (22.0%)	62/273 (22.7%)	.914*
Hypothyroidism	8/48 (16.7%)	63/266 (23.7%)	.285*
Liver failure	4/6 (66.7%)	67/308 (21.8%)	**.025** †
Cerebrovascular event	9/27 (33.3%)	62/287 (21.6%)	.164*
Renal insufficiency	12/43 (27.9%)	59/271 (21.8%)	.372*
COVID-19 (in anamnesis)	11/54 (20.4%)	60/260 (23.1%)	.665*

Surgery type	Delirium in patients with surgery n (%)	Delirium in patients without surgery n (%)	*P*
Major abdominal surgery	25/113 (22.1%)	46/201 (22.9%)	.877*
Minor abdominal surgery	7/64 (10.9%)	64/250 (25.6%)	**.012** *
Femur surgery	15/43 (34.9%)	56/271 (20.7%)	**.038** *
Spinal cord surgery	10/30 (33.3%)	61/284 (21.5%)	.140*
Other orthopedic surgeries	3/15 (20.0%)	68/299 (22.7%)	>.999†
Minor urinary surgeries	4/12 (33.3%)	67/302 (22.2%)	.479†
Major head and neck surgery	2/15 (13.3%)	69/299 (23.1%)	**.534** †
Thoracic region surgeries	1/5 (20.0%)	70/309 (22.7%)	>.999†
Other surgeries	**4/17 (23.5%**)	**67/297 (22.6%**)	>.999†

Significant results (*P* < .05) is shown in bold.

COPD = chronic obstructive pulmonary disease, COVID-19 = coronavirus disease 2019.

* Pearson chi-square.

† Fischer exact test.

General anesthesia was administered to 303 patients (96.5%), while 11 patients (3.5%) underwent regional anesthesia. Sevoflurane inhalation anesthesia was used in 295 patients (93.94%), and propofol–remifentanil total intravenous anesthesia in 8 patients (2.54%). Upon PACU admission, 46 patients (14.64%) were breathing spontaneously, whereas 268 (85.46%) were intubated. Delirium was identified in 71 patients (22.61%) using CAM-ICU-7, ICDSC, and NuDESC assessments. The hypoactive subtype was most common (n = 54; 76.05%), followed by the hyperactive subtype (n = 17; 23.94%). When comparing delirium motor subtypes, patients with hypoactive delirium (n = 54, 76.1%) had higher rates of PACU midazolam infusion (53.7% vs 17.6%, *P* = .012) and fentanyl infusion (59.3% vs 23.5%, *P* = .013) than those with hyperactive delirium (n = 17, 23.9%). The durations of midazolam (*P* = .011) and fentanyl infusions (*P* = .011), as well as total administered doses of midazolam (*P* = .015) and fentanyl (*P* = .020), were significantly higher in the hypoactive group. Additionally, indwelling catheters (25.9% vs 0.0%, *P* = .016), PACU mechanical ventilation duration (*P* = .041), and postoperative wound site infections (53.7% vs 23.5%, *P* = .049) were significantly more frequent or prolonged in hypoactive delirium cases. Conversely, PACU visual analogue scale pain scores (*P* = .028) and mean body temperature (*P* = .042) were significantly higher in the hyperactive group. No significant differences were detected between subtypes regarding age, baseline laboratory parameters, or total length of hospital stay (*P* > .05). Patients who developed delirium were significantly older than those without delirium (76 [67–82] vs 67 years [57–77]; *P* < .001), and the incidence of delirium was markedly higher among individuals aged ≥65 years (*P* < .001). Delirium was also significantly associated with a MET capacity <4 (*P* = .007), frailty according to the FRAIL index (*P* = .004), and ASA physical status III to IV (*P* = .041). Delirium occurred more frequently in patients undergoing emergency surgery (*P* = .003) and was more common following orthopedic procedures (*P* = .019), particularly femur surgery (*P* = .038). Perioperatively, delirium developed in 25% of patients who received fentanyl compared with 11.1% of those who did not (*P* = .026). Conversely, delirium was observed in 18% of patients who received remifentanil and 28.2% of those who did not (*P* = .032). No significant association was found between delirium and the type or dose of other intravenous induction agents, intraoperative analgesic medications, duration of surgery, intraoperative urine output, or intraoperative crystalloid, colloid, and blood product administration (all *P* > .05). In the PACU, there was no significant association between delirium and intubated transfer, vasopressor therapy, invasive or noninvasive ventilation support, oral intake, or fecal output (all *P* > .05). However, fentanyl infusion for postoperative sedation was significantly related to delirium; both infusion duration (*P* = .003) and total fentanyl dose (*P* = .007) were higher among patients who developed delirium, while no association was found with other opioids (*P* > .05). Delirium incidence was also significantly higher in patients receiving erythrocyte suspension (38.3% vs 18.9%; *P* = .001) or fresh frozen plasma (FFP) (39.4% vs 20.6%; *P* = .015). Crystalloid volume, erythrocyte suspension, and FFP administration during PACU stay were correlated with delirium (*P* = .027, *P* = .001, and *P* = .012, respectively; Table 3).

**Table 3:** Relationship between delirium and clinical/laboratory parameters in PACU.

Treatments in PACU	No delirium (n = 243)	Delirium present (n = 71)	*P*
Midazolam duration (h)	0 (0–6)	0 (0–12)	.255*
Midazolam amount (mg)	0 (0–12)	0 (0–14)	.503*
Fentanyl duration (h)	0 (0–3)	1 (0–12)	**.003** *
Fentanyl amount (µg)	350 (0–300)	636 (0–1050)	**.007** *
Remifentanil duration (h)	0 (0–1)	0 (0–0)	.371*
Remifentanil amount (µg)	0 (0–120)	0 (0–0)	.355*
Crystalloid solution (mL)	2180 (1550–3225)	2714 (1667–4640)	**.027** *
Colloid solution (mL)	0 (0–0)	0 (0–0)	.331*
Albumin solution (mL)	0 (0–0)	0 (0–100)	.325*
Erythrocyte suspension	37 (15.2%)	23 (32.4%)	**.002** †
Fresh frozen plasma	20 (8.2%)	13 (18.3%)	**.025** †
Erythrocyte suspension (mL)	55 (0–0)	134 (0–250)	**.001** *
Fresh frozen plasma (ml)	40 (0–1600)	107 (0–1600)	**.012** *
Average hourly fluid intake	105 (83–138)	124 (90–145)	.128*
Hourly fluid intake (mL/kg/h)	1.46 (1.13–1.97)	1.63 (1.14–2.23)	.114*
Total urine volume (mL)	1350 (850-2250)	1510 (850-3200)	.175*
Hourly urine volume	62 (43–86)	62 (36–89)	.903*
Hourly urine output (mL/kg/h)	0.85 (0.55–1.29)	0.98 (0.51–1.27)	.832*
Glucose (mg/dL)	140 (117–174)	138 (112–164)	.635*
BUN (mg/dL)	16.3 (11.7–24.7)	22.6 (16.2–34.8)	**<.001** *
Creatinine (mg/dL)	0.77 (0.58–1.04)	0.89 (0.67–1.37)	**.006** *
GFR	88 (63–104)	66 (42–88)	**<.001** *
Sodium (mmol/L)	138 (136–140)	139 (136–142)	.364*
Potassium (mmol/L)	4.14 (3.7–4.46)	4.03 (3.55–4.46)	.096*
Chlorine (mmol/L)	106 (103–109)	106 (103–110)	.743*
Calcium (mg/dL)	7.93 (7.48–8.41)	7.9 (7.47–8.21)	.334*
AST (U/L)	29 (19–49)	31 (20.7–60.5)	.294*
ALT (U/L)	20 (13–41)	18 (12.7–31.2)	.481*
Albumin (g/dL)	2.94 (2.64–3.34)	2.69 (2.47–2.93)	**<.001** *
Total bilirubin (mg/dL)	0.75 (0.59–1.22)	0.85 (0.66–1.34)	.148*
Direct bilirubin (mg/dL)	0.15 (0.1–0.31)	0.21 (0.11–0.41)	**.024** *
CRP (mg/L)	58.5 (17.4–161.2)	152 (58.4–211.2)	**<.001** *
CRP/albumin ratio	20.2 (5.9–57.1)	55.5 (23.5–83.2)	**<.001** *
INR	1.06 (0.99–1.15)	1.15 (1.02–1.25)	**.001** *
Hgb (g/dL)	10.5 (9.4–12.2)	9.6 (8.3–10.9)	**<.001** *
Htc (%)	32.2 (28.7–37)	28.9 (25.4–31.3)	**<.001** *
WBC (10^3^/µL)	11.1 (8.3–14.6)	11.2 (8.9–15.2)	.392*
PLT (10^3^/µL)	228 (175–297)	225 (149–297)	.388*
Neutrophil/lymphocyte ratio	11.8 (7–18.8)	12.3 (7.6–17.2)	.677*
pH	7.38 (7.35–7.42)	7.38 (7.32–7.43)	.397*
PaCO_2_ (mm Hg)	38.6 (34.9–42.1)	37.1 (34.1–41.7)	.364*
PaO_2_ (mm Hg)	110 (88.7–141)	112 (83.7–156.5)	.843*
SaO_2_ (%)	98.4 (97.2–99)	98 (96.9–98.9)	.475*
HCO_3_ (mm Hg)	23 (21.4–25.3)	22.6 (19.4–25.3)	.110*
BE	−1.65 (−3.7 to −1.2)	−1.2 (−5.6 to −2.6)	.594*
Lactate (mmol/L)	1.2 (0.9–1.9)	1.35 (1–1.65)	.614*

Significant results (*P* < .05) is shown in bold.

ALT = alanine aminotransferase, AST = aspartate aminotransferase, BE = base excess, BUN = blood urea nitrogen, CRP = C-reactive protein, GFR = glomerular filtration rate, HCO_3_ = bicarbonate, Hct = hematocrit, Hgb = hemoglobin, INR = international normalized ratio, PaCO_2_ = partial pressure of carbon dioxide, PACU = postanesthesia care unit, PaO_2_ = partial pressure of oxygen, PLT = platelet count, SaO_2_ = arterial oxygen saturation, WBC = white blood cell count.

* Mann–Whitney *U* test.

† Pearson chi-square.

Laboratory evaluations showed that delirium was associated with higher blood urea nitrogen (*P* < .001), lower creatinine (*P* = .006), higher direct bilirubin (*P* = .024), higher CRP (*P* < .001), higher CRP/albumin ratio (*P* < .001), elevated international normalized ratio (INR) (*P* = .001), reduced GFR (*P* < .001), lower albumin (*P* < .001), lower hemoglobin (*P* < .001), and lower hematocrit. Arterial blood gas values closest to the delirium assessment were not associated with delirium (Table 3). Additionally, there was no association between the presence or number of drains and catheters (epidural, nasogastric, urinary, etc) and delirium (*P* > .05).

In the multivariable logistic regression model (Table 4), 5 variables remained independently associated with postoperative delirium: age ≥65 years (OR: 3.08; 95% CI: 1.10 to 8.63; *P* = .032), higher PACU pain scores (OR: 1.28; 95% CI: 1.03 to 1.59; *P* = .025), lower hematocrit (OR: 0.89; 95% CI: 0.82 to 0.98; *P* = .014), decreased GFR (OR: 0.98; 95% CI: 0.97 to 1.00; *P* = .014), and longer fentanyl infusion duration in the PACU (OR: 1.09; 95% CI: 1.01 to 1.17; *P* = .025). The relatively wide confidence intervals observed for certain dichotomous predictors (e.g., age ≥65 years) reflect lower subgroup sample distribution and variance inherent to dichotomized clinical variables. The model demonstrated good discrimination (AUC = 0.849) and adequate calibration (Hosmer–Lemeshow *P* = .31).

**Table 4:** Multivariate logistic regression analysis for factors associated with postoperative delirium.

			95% confidence interval	
Predictor	*P*	Odds ratio	Lower	Upper
Age ≥65 yr (yes = 1, no = 0)	**.032** *	3.08	1.100	8.633
Glomerular filtration rate (GFR)	**.014** *	0.98	0.967	0.996
Hematocrit (Hct)	**.014** *	0.89	0.815	0.978
Visual analogue scale (VAS) pain score	**.025** *	1.28	1.031	1.589
Duration of fentanyl infusion in PACU (h)	**.025** *	1.09	1.010	1.174

Candidate variables entered into the initial screening model but eliminated during stepwise selection included: ASA physical status, FRAIL score, MET capacity, hypertension, emergency surgery, hip surgery, intraoperative fentanyl, CRP, albumin, direct bilirubin, INR, blood transfusions, PACU mechanical ventilation duration, and PACU length of stay.

Bold values indicate statistically significant independent predictors (*P* < .05).

ASA = American Society of Anesthesiologists, AUC = area under the curve, CI = confidence interval, CRP = C-reactive protein, EPV = events per variable, FRAIL = fatigue, resistance, ambulation, illnesses, and loss of weight index, GFR = glomerular filtration rate, Hct = hematocrit, INR = international normalized ratio, MET = metabolic equivalent of task, OR = odds ratio, PACU = postanesthesia care unit, VAS = visual analogue scale.

* Model diagnostics: Backward stepwise elimination method was applied (Hosmer–Lemeshow test *P* = .31, AUC = 0.849, events per variable ratio = 14.2).

Delirium was significantly associated with longer PACU stay (*P* = .016), prolonged duration of mechanical ventilation in the PACU (*P* = .006), and higher postoperative pain scores (*P* < .001). Patients with delirium also had significantly longer ICU stays (*P* < .001) and total hospital stays (*P* = .009) (Table 5). Furthermore, delirium was associated with higher rates of postoperative complications, including wound infection (*P* = .026), sepsis (*P* < .001), pulmonary complications (*P* = .015), pneumonia (*P* = .046), renal failure (*P* < .001), ICU admission (*P* < .001), and mortality (*P* < .001) (Table 5).

**Table 5:** Association of delirium with PACU ventilation support, patients’ vital values, length of hospitalization and postoperative complications.

Variables	No delirium (n = 243)	Delirium present (n = 71)	*P*
Length of stay in PACU (h)	22 (17–31)	26 (18–40)	**.016** *
Mechanical ventilator duration (h)	5 (2–13)	9 (4–18)	**.006** *
Length of hospitalization (d)	12 (7–19)	14 (10–28)	**.009** *
Length of intensive care unit hospitalization (d)	0.5 (0–29)	2.3 (0–42)	**<.001** *
Duration of noninvasive mechanical ventilation (h)	0 (0–0)	0 (0–0)	.356*
Mean arterial pressure (mm Hg)	95 (87–101)	92 (85–101)	.173*
Average pulse rate (bpm)	86 (74–97)	88 (80–100)	.155*
Highest temperature (°C)	36.3 (36.2–36.6)	36.4 (36.2–36.7)	.077*
Visual pain scoring	2 (0–3)	3 (1–5)	**<.001** *
Wound site infection	78 (32.1%)	33 (46.5%)	**.026** †
Sepsis	21 (8.6%)	21 (29.6%)	**<.001** †
Pulmonary complications	63 (25.9%)	29 (40.8%)	**.015** †
Pneumonia	21 (8.6%)	12 (16.9%)	**.046** †
Renal insufficiency	32 (13.2%)	22 (31%)	**<.001** †
Intensive care hospitalization	16 (6.6%)	16 (22.5%)	**<.001** †
Mortality	12 (4.9%)	14 (19.7%)	**<.001** †

Significant results (*P* < .05) is shown in bold.

BPM = beat per minute, PACU = postanesthesia care unit.

* Mann–Whitney *U* test.

† Fisher exact test.

## 4. Discussion

Delirium is the most common neurological complication observed in the postoperative period. Bilge et al^[6]^ reported an incidence of 18.4% in a prospective cohort of 250 adult patients, while a meta-analysis by Bruce et al^[11]^ of 26 studies described a wide range from 4% to 53.3% in hip fracture patients and 3.6% to 28.3% among elective surgical cohorts. The incidence of delirium in our study (22.6%) is consistent with these previous reports.

Advanced age has been repeatedly identified as one of the strongest predictors of postoperative delirium. Both Hua et al^[12]^ and Xiao-Hong Liu et al^[13]^ demonstrated age as an independent risk factor, and our multivariable logistic regression analysis similarly showed that age ≥65 years was independently associated with an increased risk. Age-related reductions in physiological reserve, impaired neurotransmitter regulation, and diminished cerebral tolerance to perioperative stress likely contribute to this vulnerability.

In a meta-analysis, Hua et al^[12]^ also identified ASA physical status III to IV as an independent risk factor for delirium, and Wittmann et al^[14]^ demonstrated a higher incidence among patients with MET capacity <4 in a cohort of 976 cases. Our findings align with this evidence, indicating that patients with severe comorbidities and reduced functional capacity are at heightened risk. These individuals frequently exhibit compromised metabolic adaptation and reduced perfusion reserve, predisposing them to cerebral hypoxia and metabolic stress under surgical conditions.

Frailty has similarly been shown to predispose patients to postoperative delirium. Frost et al^[15]^ demonstrated that higher frailty index scores increased delirium risk among 2566 surgical patients. Consistent with prior evidence, our results reinforce the concept that frailty – a state characterized by sarcopenia, diminished energy metabolism, and chronic inflammation – reflects systemic vulnerability that heightens susceptibility to perioperative cognitive dysfunction.

Several comorbidities have been linked to delirium. Wu et al^[16]^ identified hypertension and chronic obstructive pulmonary disease as significant risk factors, while Schenning et al^[17]^ reported associations with heart failure, diabetes mellitus, renal disease, and vascular pathologies. Denk et al^[18]^ found liver disease to be contributory. In our cohort, patients with chronic hypertension may have experienced perioperative blood pressure fluctuations, ischemic episodes, or microembolic events, resulting in intermittent cerebral hypoperfusion and contributing to delirium. Although ASA III to V status, FRAIL >3, MET <4, hypertension, and hip surgery did not remain significant in the multivariable model, this is likely attributable to collinearity with age, which persisted as an independent predictor.

Consistent with the findings of Ansaloni et al^[19]^ and Chaiwat et al,^[20]^ our study demonstrated a higher delirium incidence among patients undergoing emergency surgery. This pattern may reflect insufficient preoperative optimization, hemodynamic instability, prolonged fasting, and heightened inflammatory activation associated with urgent procedures.

In our cohort, patients receiving perioperative remifentanil exhibited lower delirium incidence – a finding similar to Radtke et al^[21]^ – nd this may reflect the preferential use of fentanyl in frail individuals with limited hemodynamic reserve. Existing literature shows no consensus on the association between opioids and delirium. Fong et al^[22]^ reported that meperidine increased delirium risk in elderly patients, whereas no association was found for morphine, fentanyl, or hydromorphone. Sica et al^[23]^ suggested that delirium linked to opioids may reflect uncontrolled pain rather than opioid exposure itself. In our study, delirium was not associated with tramadol, meperidine, or morphine use in the intraoperative or PACU periods. These results support the hypothesis that pain-related sympathetic activation, rather than exposure to a specific opioid, plays a greater role in postoperative cognitive dysfunction.

Blood transfusion has been implicated in delirium risk. Bramley et al^[24]^ identified intraoperative blood product administration as a risk factor, whereas van der Zanden et al^[25]^ noted that hemoglobin levels <6 mg/dL increased delirium risk and suggested that transfusion may be protective under profound anemia. Chou et al^[26]^ reported higher delirium incidence in anemic patients receiving transfusion, with no significant increase in non-anemic recipients. Zhao et al,^[27]^ in a meta-analysis of 3,767,761 patients, concluded that both low preoperative and postoperative hemoglobin levels contribute to delirium development. In line with these findings, our multivariable analysis identified low hematocrit as an independent risk factor. Physiologically, an acute reduction in hematocrit directly compromises arterial oxygen content and cerebral oxygen delivery. Because the aging or physiologically fragile brain possesses restricted metabolic reserve, acute perioperative anemia precipitates localized cerebral hypoxia, microvascular oxidative stress, impaired neurovascular coupling. The higher rates of delirium in patients receiving erythrocyte suspension or FFP may also reflect transfusion-related immunologic effects or inadequate tissue oxygenation.

Casault et al^[28]^ demonstrated that midazolam- and fentanyl-based sedation strategies were associated with higher delirium risk and prolonged intubation in ICU patients. Consistent with these findings, our study identified prolonged intubation and intravenous sedative infusions in the PACU – particularly fentanyl infusion duration – as independent predictors of delirium. However, this finding requires nuanced clinical interpretation regarding confounding by indication. In our perioperative workflow, continuous fentanyl infusions were specifically prioritized over ultra-short-acting opioids (such as remifentanil) for patients anticipated to have delayed extubation, prolonged PACU recovery, or an extended need for mechanical ventilation and endotracheal tube tolerance. Consequently, fentanyl usage served as an implicit marker for a physiologically more complex patient cohort requiring prolonged airway control, rather than merely representing an escalation in response to uncontrolled acute pain. Beyond this selection bias, sedative agents may contribute through disruption of sleep–wake cycles, suppression of cortical activity, and delayed cognitive recovery. Furthermore, continuous exposure to synthetic opioids can directly induce central neurotoxicity by altering cholinergic neurotransmission, fluctuating sedation depth, and impairing cerebral perfusion matching in vulnerable PACU patients.

Renal dysfunction has been associated with delirium in several studies. Park et al^[29]^ showed that elevated blood urea nitrogen and creatinine levels increased delirium risk, and our findings similarly demonstrated a higher incidence among patients with reduced GFR (OR: 0.982; 95% CI: 0.967 to 0.996; *P* = .014). Impaired baseline renal function reflects underlying systemic microvascular endothelial disease and compromises the clearance of uremic toxins and pro-inflammatory mediators. The systemic accumulation of these metabolites increases blood–brain barrier permeability, triggers microglial activation, and disrupts central cholinergic signaling. Furthermore, patients with reduced GFR exhibit impaired cerebral autoregulation, rendering them particularly susceptible to intermittent cerebral hypoperfusion and cognitive breakdown under transient perioperative hemodynamic fluctuations.

Hepatic dysfunction has also been implicated. Liu et al^[30]^ identified hypoalbuminemia as a risk factor, while Aldemir et al^[31]^ reported elevated bilirubin levels among ICU patients with delirium. Consistent with Guo et al,^[32]^ our study showed higher delirium incidence in patients with lower albumin and elevated bilirubin, supporting the role of impaired hepatic metabolism and systemic inflammation. As hypoalbuminemia also reflects malnutrition and reduced intravascular volume, these factors may further increase delirium risk.

Inflammatory markers have consistently been associated with delirium. Sun et al^[33]^ and Vasunilashorn et al^[34]^ reported that elevated postoperative CRP levels predict delirium in colorectal and noncardiac surgeries, respectively. Kim et al^[35]^ similarly linked higher CRP-to-albumin ratios with delirium. In agreement, our findings showed that both CRP and CRP/albumin ratio were elevated in patients with delirium, supporting the hypothesis that perioperative inflammatory stress is a central contributor.

Coagulation abnormalities may also reflect underlying systemic pathology. Gutowski et al^[36]^ found prolonged INR to be associated with delirium in ICU patients with COVID-19 pneumonia. Abazid et al^[37]^ similarly reported elevated INR among patients with delirium. In our cohort, higher INR likely reflected hepatic dysfunction or systemic inflammation, both of which may impair cerebral microcirculation and promote delirium.

Pain has been recognized as a modifiable risk factor. Vaurio et al^[38]^ demonstrated that higher postoperative visual analogue scale scores predicted delirium, and our multivariable analysis confirmed this association. Pain-related sympathetic activation, sleep disruption, and psychological distress may destabilize cognition. As suggested by Daoust et al,^[39]^ our findings support the notion that pain severity – rather than opioid dosage – is a more direct driver of delirium.In this context, implementation of multimodal non-opioid analgesic strategies represents a crucial perioperative approach. Combining regional anesthesia techniques (e.g., peripheral nerve blocks or epidural analgesia) with non-opioid systemic agents – such as intravenous acetaminophen, nonsteroidal anti-inflammatory drugs (NSAIDs), or dexmedetomidine – can effectively suppress surgical nociceptive input while significantly sparing opioid consumption. By mitigating severe pain-induced sympathetic surge and central neuroinflammation without imposing additional GABAergic or anticholinergic sedative burden, multimodal analgesia holds clinical potential to break the vicious cycle between uncontrolled postoperative pain and opioid-induced cognitive impairment in vulnerable PACU patients.

Finally, consistent with the meta-analysis by Salluh et al^[40]^ involving 16,595 patients, delirium in our cohort was associated with prolonged mechanical ventilation, longer PACU, ICU, and hospital stays, and higher mortality. These results reinforce delirium as both an acute neurocognitive disorder and a prognostic marker of adverse postoperative outcomes. Prior studies have also linked delirium to infectious complications. Zukowska et al^[41]^ observed higher rates of pneumonia and wound infections after cardiac surgery, while Ahn et al^[42]^ reported increased postoperative and aspiration pneumonia in elderly hip-fracture patients. Sepsis-associated encephalopathy has been described by Piva et al^[43,44]^ as a frequent manifestation of systemic infection, and Jackel et al^[45]^ found delirium in 60% of ICU patients with acute kidney injury. In our study, wound infection, sepsis, pneumonia, and renal failure were more common among patients with delirium, supporting the close interplay between systemic inflammation, infection, and cerebral dysfunction.

## 5. Conclusion

In conclusion, postoperative delirium was common among patients admitted to the PACU and was associated with advanced age, frailty, reduced functional capacity, anemia, renal and hepatic dysfunction, and severe postoperative pain. Delirium was also linked to prolonged PACU, ICU, and hospital stays, as well as higher rates of complications and mortality. Because most cases were of the hypoactive subtype – which is easily overlooked – structured and repeated screening during the early postoperative period may improve detection.

Although this study was not designed to evaluate targeted prevention strategies, the findings – together with existing evidence – suggest that several modifiable perioperative factors warrant closer attention in routine practice, including adequate pain management, judicious use of sedatives, hemodynamic stabilization, and timely correction of anemia and metabolic disturbances. Future multicenter studies are needed to confirm these associations and to determine whether optimization of these domains can effectively reduce the burden of postoperative delirium.

## 6. Limitations

This study has several limitations. First, it was conducted at a single tertiary university hospital, which may limit the generalizability of the findings to institutions with different patient populations, perioperative workflows, and postoperative care pathways. External validation in multicenter cohorts with diverse surgical case mixes is therefore warranted.

Second, although delirium was assessed using validated tools (CAM-ICU-7, ICDSC, and NuDESC), some degree of observer variability is unavoidable.

Third, baseline neurocognitive status was evaluated via preexisting medical history, health records, and caregiver reports rather than routine formal objective screening tools (such as the mini-mental state examination or Montreal cognitive assessment). Consequently, subtle or undiagnosed baseline mild cognitive impairment may not have been fully identified, representing a potential unmeasured baseline confounder.

Fourth, processed electroencephalographic depth of anesthesia monitoring (such as bispectral index ) was not routinely used for all participants, which may have allowed unmeasured variations in anesthetic depth or burst suppression episodes to act as residual intraoperative confounders. Additionally, while surgical and anesthetic durations were prospectively evaluated, heterogeneity in surgical complexity across procedures represents a potential source of residual variation despite multivariable adjustment.

Additionally, certain perioperative interventions – such as transfusions, opioids, and sedative infusions – were more frequently administered to clinically unstable or symptomatic patients, raising the possibility of confounding by indication. As an observational study, the design cannot fully separate the effects of treatment from the underlying severity of illness. Future research employing causal inference techniques, such as propensity-score methods or marginal structural models, may better elucidate these relationships.

Fifth, sample size constraints within specific subcategories resulted in relatively wide CIs for certain multivariable estimates (such as age ≥65 years and intraoperative fentanyl administration), indicating that larger prospective multicenter cohorts are required to obtain more precise effect-size estimations.

Sixth, the study was not preregistered in an observational research registry, which introduces a potential risk of selective reporting.

Finally, delirium assessments were limited to the PACU stay. Episodes occurring after transfer to the ward or ICU may have been missed, potentially leading to underestimation of the true postoperative delirium incidence.

## Acknowledgments

The authors thank the nursing and medical staff of the postanesthesia care unit at Dokuz Eylül University Hospital for their assistance during data collection.

## Author contributions

**Conceptualization:** Muhammed Emin Yildirim, Volkan Hanci, Duriye Gul Inal.

**Data curation:** Muhammed Emin Yildirim, Hale Aksu.

**Formal analysis:** Muhammed Emin Yildirim.

**Investigation:** Muhammed Emin Yildirim, Duriye Gul Inal.

**Methodology:** Volkan Hanci, Duriye Gul Inal, Sule Ozbilgin.

**Project administration:** Volkan Hanci.

**Resources:** Volkan Hanci, Sule Ozbilgin.

**Software:** Muhammed Emin Yildirim

**Supervision:** Volkan Hanci.

**Validation:** Duriye Gul Inal, Semih Kucukguclu.

**Visualization:** Muhammed Emin Yildirim, Volkan Hanci.

**Writing – original draft:** Muhammed Emin Yildirim.

**Writing – review & editing:** Muhammed Emin Yildirim, Volkan Hanci, Duriye Gul Inal, Hale Aksu, Sule Ozbilgin, Semih Kucukguclu.
